# Long noncoding RNAs in neuronal-glial fate specification and oligodendrocyte lineage maturation

**DOI:** 10.1186/1471-2202-11-14

**Published:** 2010-02-05

**Authors:** Tim R Mercer, Irfan A Qureshi, Solen Gokhan, Marcel E Dinger, Guangyu Li, John S Mattick, Mark F Mehler

**Affiliations:** 1 Institute for Molecular Bioscience, University of Queensland, 306 Carmody Road, Brisbane, QLD 4072, Australia; 2 Institute for Brain Disorders and Neural Regeneration, Albert Einstein College of Medicine, Bronx, New York, NY 10461, USA; 3 Department of Neurology, Albert Einstein College of Medicine, Bronx, New York, NY 10461, USA; 4 Department of Psychiatry and Behavioral Sciences, Albert Einstein College of Medicine, Bronx, New York, NY 10461, USA; 5 Einstein Cancer Center, Albert Einstein College of Medicine, Bronx, New York, NY 10461, USA; 6 Rose F Kennedy Center for Research on Intellectual and Developmental Disabilities, Albert Einstein College of Medicine, Bronx, New York, NY 10461, USA

## Abstract

**Background:**

Long non-protein-coding RNAs (ncRNAs) are emerging as important regulators of cellular differentiation and are widely expressed in the brain.

**Results:**

Here we show that many long ncRNAs exhibit dynamic expression patterns during neuronal and oligodendrocyte (OL) lineage specification, neuronal-glial fate transitions, and progressive stages of OL lineage elaboration including myelination. Consideration of the genomic context of these dynamically regulated ncRNAs showed they were part of complex transcriptional loci that encompass key neural developmental protein-coding genes, with which they exhibit concordant expression profiles as indicated by both microarray and *in situ *hybridization analyses. These included ncRNAs associated with differentiation-specific nuclear subdomains such as *Gomafu *and *Neat1*, and ncRNAs associated with developmental enhancers and genes encoding important transcription factors and homeotic proteins. We also observed changes in ncRNA expression profiles in response to treatment with trichostatin A, a histone deacetylase inhibitor that prevents the progression of OL progenitors into post-mitotic OLs by altering lineage-specific gene expression programs.

**Conclusion:**

This is the first report of long ncRNA expression in neuronal and glial cell differentiation and of the modulation of ncRNA expression by modification of chromatin architecture. These observations explicitly link ncRNA dynamics to neural stem cell fate decisions, specification and epigenetic reprogramming and may have important implications for understanding and treating neuropsychiatric diseases.

## Background

It has recently become evident that the majority of the mammalian genome is transcribed in a developmentally regulated manner producing tens of thousands of interleaved short and long ncRNAs [1-4]. Long ncRNAs, defined as greater than 200 nt in length [5], exhibit specific temporal and spatial expression patterns in mouse brain [6], embryonic stem cell differentiation [7] and T-cell differentiation [8]. NcRNAs are also implicated in neural developmental events and in the pathogenesis of neuropsychiatric diseases [9]. These long ncRNAs are being shown to have a wide range of functions, including roles in the regulation of chromosomal architecture and dynamics, transcription, post-transcriptional processing, RNA editing, RNA trafficking and organelle biogenesis [4,10-14]. One major emergent function of long ncRNAs is their ability to modulate the epigenetic status of nearby protein-coding genes by recruiting chromatin activator or repressor complexes [4,12,15].

Here we present the first systematic examination of long ncRNA expression profiles during differentiation of embryonic forebrain-derived neural stem cells (NSCs). We focused on a subset of cortical GABAergic neurons (GABANs) and oligodendrocytes (OLs) that are initially specified from sonic hedgehog (Shh)-responsive, *Nkx2.1*-expressing bipotent neuronal-OL progenitor (N/OP) species within the ventromedial forebrain. We have previously reported that these N/OPs undergo long-distance tangential migration to cerebral cortex where they sequentially give rise to GABANs and OLs [16-21]. We employed custom designed microarrays to examine expression of noncoding and protein-coding transcripts during these neural developmental transitions, finding dynamic expression of ncRNAs associated with key neural development genes. We also observed differential expression of specific ncRNAs after treatment of oligodendrocyte progenitors (OLPs) with trichostatin A (TSA), a histone deacetylase (HDAC) inhibitor that prevents progression of OLPs into post-mitotic oligodendrocytes (PMOs) by suppressing oligodendrocyte-specific genes and inducing epigenetic reprogramming [22-26]. These developmental changes underscore the importance of epigenetic regulation in the elaboration of neural cell types and imply functional relationships between chromatin remodeling and selective deployment of ncRNAs.

## Results

**Figure 1:**
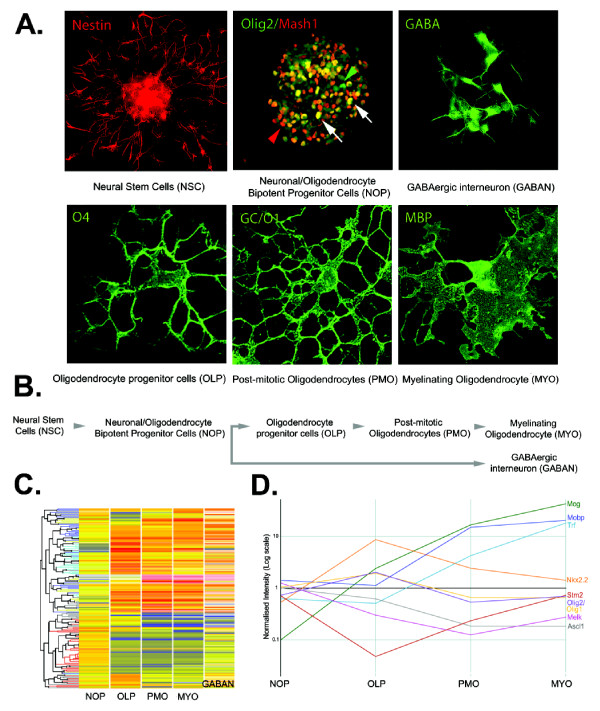
**Profiles of gene expression during neural stem cell-mediated neural lineage elaboration**. **(A) **Immunofluorescence micrographs of cellular expression patterns of lineage markers and bHLH transcription factors during progressive GABAergic neuronal and OL lineage elaboration from N/OPs: NSCs are labeled with nestin (TRITC) only, while N/OPs (white arrows) express both Olig2 (FITC, green arrow head) and Mash 1 (TRITC, red arrow head). GABANs expressed GABA (FITC). OLPs are stained with O4 (FITC), while PMOs and MYOs are identified by the expression of GC/O1 (FITC) and MBP (FITC), respectively. **(B) **Schematic flowchart illustrating the stages of oligodendrocyte and GABAergic neuron lineages analyzed within this study. (**C**) Tree-plot showing expressed ncRNAs clustered according to differential expression during microarray analysis. **(D) **Expression of mRNA marker genes with well characterized roles in GABAergic neuronal and progressive stages of OL lineage elaboration as determined by microarray.

We conducted gene expression profiling using a custom-designed microarray platform to examine protein-coding and noncoding transcript expression accompanying neural stem cell-mediated fate restriction (bipotent progenitor cells: N/OPs), neural lineage specification (GABAergic neurons: GABANs and oligodendrocyte progenitors: OLPs), neuronal-glial fate switching (GABANs → OLPs), and neural lineage maturation (post-mitotic oligodendrocytes: PMOs and myelinating oligodendrocytes: MYOs) (Figure 1). In total, we found 31% (4,616 out of 14,827) of probes targeting protein-coding transcripts exhibited expression above background and that 2366 (16%) of these were significantly differentially expressed (B-statistic > 1) at one or more developmental stages compared to NSCs [Additional file 1].

We found that 9% (332 out of 3,659) of probes targeting ncRNAs were expressed above background, of which 169 (5%) were significantly differentially expressed (B-statistic > 1; Figure 1) at one or more developmental stages compared to the NSC control [Additional file 2). These data reveal that a smaller percentage of ncRNAs are differentially expressed relative to protein-coding mRNAs. While this difference may be due to relatively poor annotation of the long noncoding transcriptome, it is consistent with previous reports in other developmental models [1,27] and may reflect the fact that ncRNAs are primarily regulatory in nature requiring only subtle changes in expression to carry out their functional roles [28]. We also validated our microarray findings with QRT-PCR and found an 87% correlation between the gene expression profiles from the microarray data and QRT-PCR results [Additional file 3].

### Genomic analysis of expressed ncRNAs

Long ncRNAs are often organized as part of complex interleaved transcriptional networks that include protein-coding genes [1,2]. A number of studies have shown that the association of long noncoding transcription with protein-coding genes has functional relevance, often with the ncRNA regulating expression of its protein-coding counterpart via epigenetic modifications and/or transcriptional co-activation/repression [14,15,29-31]. Therefore, we identified all ncRNAs expressed in our developmental models that are transcribed antisense to, bi-directionally, or from within the introns of overlapping or adjacent protein coding genes [Additional file 4]. In addition, we identified pairs of protein-coding RNAs and ncRNAs whose genomic loci are positionally conserved between mouse and human [Additional file 4] because this genomic conservation may also indicate a functional relationship between the two transcripts. In many cases, the associated protein-coding genes were critical neuro-developmental genes. As an illustrative example, the downregulation of a ncRNA (*AK053922*) is associated with the *Gli3 *locus in all progeny of N/OPs, whereas *Gli3 *was upregulated in N/OPs but downregulated in all progeny of N/OPs. This relationship may be of significant interest given that *Gli3 *has been shown to act as a bifunctional transcriptional switch that can either repress or activate Shh signaling [32] to help specify distinct neuronal cell types [33].

Many ncRNAs contain conserved secondary structures that are crucial to their function [34]. These conformational features may be bound by proteins [35], processed into smaller regulatory ncRNAs [36] or even fulfill intrinsic catalytic functions [37]. To provide support for the proposed functionality of the expressed ncRNAs, we looked for evidence of RNA secondary structures within differentially expressed ncRNA transcripts using the folding program RNAz [34]. We found 74 expressed ncRNAs that contained high confidence predicted conserved secondary structures (RNAz P > 0.9) [Additional file 5] such as stem-loops [Additional file 6], known to be present in miRNA precursors, small nucleolar RNAs (snoRNAs) and other RNAs with previously characterized functions.

We then combined these genomic analyses to identify candidate ncRNAs for further functional analysis. These are discussed in more detail below in the context of neuronal and oligodendroglial lineage commitment, neuronal-glial lineage switching and progressive stages of oligodendrocyte lineage elaboration.

### Lineage restriction of neural stem cells (NSCs) into bipotent neuronal/oligodendrocyte progenitors (N/OPs)

A subset of nestin-immunoreactive NSCs within the ventral forebrain give rise to bipotent N/OPs following exposure to a gradient of Shh signaling [17,20] which are characterized by expression of two bHLH transcription factors (*Olig2 *and *Mash1*) and their potential to give rise to GABANs and OLs but not astrocytes [16,20]. Our microarray analysis revealed that the initial fate restriction of regional forebrain NSCs to bipotent N/OPs was associated with relatively modest changes in gene expression profiles, possibly reflecting the relative similarities between these developmental cell types. Nonetheless, there were differences in factors that may be important for promoting NSC lineage restriction and abrogating self-renewal and maintenance programs, including 65 upregulated and 167 downregulated mRNAs [Additional file 1]. Not surprisingly, some genes previously implicated in neurogenesis and gliogenesis exhibited dynamic changes in mRNA expression including transcription factors (52% of upregulated genes; p < 0.01) and cell-surface receptor linked signal transduction pathways (21% of downregulated genes; p < 0.01). For example, we found upregulated mRNAs included genes such as *Mash1*, an important transcription factor that promotes lineage restriction of NSCs into N/OPs [38-40].

We also identified dynamically expressed ncRNAs that may fulfill functional roles in NSC fate restriction. Similar to mRNAs, relatively few changes in ncRNA expression were observed during the transition from NSCs to N/OPs, with 17 ncRNAs exhibiting differential expression (8 upregulated and 9 downregulated) [Additional file 2]. These included a previously described ncRNA, *Gomafu*, shown to be localized to a novel subnuclear domain in a distinct subset of differentiating neurons in the mouse nervous system [41], which was exclusively downregulated in N/OPs but upregulated in all subsequent stages of OL lineage specification and maturation [Additional file 7]. Furthermore, *in situ *hybridization (ISH) in the adult mouse brain showed *Gomafu *expression in specific neuronal populations and nuclear localization within Purkinje cells [Additional file 7]. Our results show that *Gomafu *is not only expressed during neurogenesis but also during OL lineage specification.

**Figure 2:**
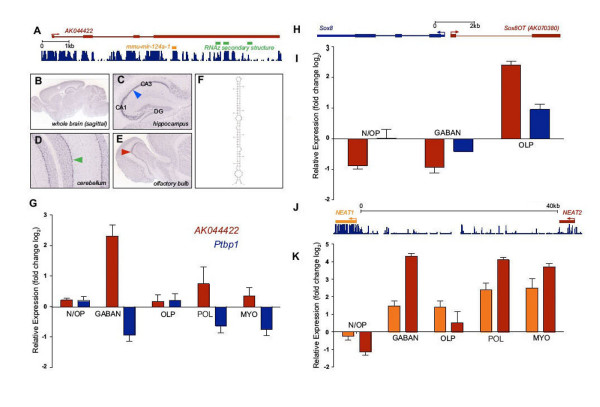
**Detailed examples of ncRNAs expressed during oligodendrocyte differentiation**. (A-F) Expression of the ncRNA *AK044422 *during GABAN and OL differentiation. (A) Genomic context of the ncRNA *AK044422 *transcript (red), *miR-124a *(orange), RNA secondary structures as predicted by RNAz [101] (green). (B-E) *In situ *hybridization of adult mouse brain sagittal sections for *AK044422 *transcript. (B) *AK044422 *is expressed broadly throughout the adult mouse brain. (C) *AK044422 *exhibits an enriched expression in the *Cornu Ammonis *subfields (CA1-3; blue arrow) (D) Detail of cerebellum shows *AK044422 *exhibits enriched expression in Purkinje neurons (green arrow). (E) Detail of olfactory bulb shows enriched expression in the mitral layer (red arrow). *Images courtesy of *(*Allen Brain Atlas*, http://brain-map.com). (F) Example of RNA secondary structure prediction within *AK044422 *transcript as rendered by CONTRAfold [99]. (G) Expression of *AK044422 *(red) and *Ptbp1 *(blue) according to microarray analysis (expression is relative to NSCs and error bars show standard deviation). (H) Genomic context of *Sox8 *(blue) and ncRNA *Sox8OT *transcript (*AK070380*; red). (I) Expression of *Sox8OT *(red) and *Sox8 *(blue) according to microarray analysis. (J) Genomic context of the ncRNAs *Neat1 *(orange) and *Neat2 *(dark red) and histogram of vertebrate conservation (dark blue). (K) Expression of *Neat1 *(orange) and *Neat2 *(red) according to microarray analysis.

A number of ncRNAs are significantly upregulated only during lineage restriction of NSCs, including the *small nucleolar RNA (snoRNA) host gene 1 (Snhg1) and 10 (Snhg10)*, an expression profile supported by the restricted neuronal expression of *Snhg10 *in whole brain ISH [Additional file 8]. *Snhg1 *and *Snhg10 *host a group of C/D box snoRNAs that serve as guides for the site-specific 2'-O-methylation of ribosomal RNAs. Furthermore, two identified ncRNA species, *Neat1 *(also known as *Men ε/β*) and *Neat2*, are downregulated in N/OPs and broadly upregulated in their neuronal and glial progeny (Figure 2). *Neat1 *and *Neat2 *associate with nuclear paraspeckles and adjacent SC35 speckles, respectively; domains where transcription and co-transcriptional pre-mRNA processing occur [42]. These nuclear domains have been associated with modulation of cell growth and differentiation [43,44], and *Neat1 *(*Men ε/β*) has been recently shown to be induced upon cell differentiation and to be required for the structural integrity of paraspeckles [45-47].

### Specification of GABAergic neurons (GABANs)

Bipotent N/OPs can differentiate into either GABANs or OLPs in response to different neural cytokines [20]. We therefore examined changes in gene expression during the elaboration of GABANs to identify mRNAs and ncRNAs that may be involved in this type of neuronal lineage specification. We observed upregulation of 483 mRNAs, which were enriched for genes involved in nervous system development (17.3%; p < 0.03), neuronal function (9.88%, p < 0.05) and neurogenesis (10.67%, p < 0.05). We also identified 330 downregulated mRNAs [Additional file 1]. Interestingly, these downregulated mRNAs were enriched for predicted targets of *let-7b *(13.2% p < 0.01) and *mir-135 *(11.3%; p < 0.01), possibly being subject to repression by miRNAs. Indeed, both *let-7b *and *mir-135 *have previously been implicated in promoting neurogenesis [48,49]. In addition, we identified 56 ncRNAs that are upregulated during GABAN neurogenesis, including a number of examples such as *Gtl2*, *Rian, Evf2*, and *Copg2as *that were previously shown to exhibit neuron-enriched expression profiles in the adult mouse brain [6]. In contrast, we identified only 8 ncRNAs that were downregulated during GABAergic neurogenesis [Additional file 2].

We distinguished ncRNAs that may be involved specifically in GABAN lineage commitment by selecting ncRNAs upregulated during GABAN differentiation but downregulated during OL differentiation [Additional file 9]. We identified 4 ncRNAs, including an intergenic ncRNA transcript *AK044422 *that overlaps a highly conserved brain-specific miRNA, *miR-124a*. *MiR-124a *accounts for nearly half of all brain miRNAs [50] (Figure 2) and helps to regulate neuronal specification and differentiation [51-53]. One mechanism by which *miR-124 *was shown to promote neuronal differentiation is by repression of *Ptbp1*, a negative regulator of alternative splicing, resulting in activation of neuron-specific alternative splicing events [53]. Indeed, we found that *Ptbp1 *and *AK044422 *exhibited complementary expression profiles. However, the *AK044422 *transcript may itself function intrinsically as a full-length ncRNA, rather than simply acting as a precursor. The *AK044422 *transcript is subject to post-transcriptional modifications including splicing and polyadenylation, contains a number of high confidence predicted stem-loop structures (Fig 2.), and is highly conserved with homologous transcripts present in humans (*AK091593, AL832535*) and rats (*BF402302*). Indeed, the *AK044422 *transcript was recently identified in a genome wide screen for functional lncRNAs, termed lincRNAs, that exhibit a distinct chromatin signature indicative of transcriptional regulation and high levels of expression [54]. Furthermore, ISH in adult mouse brain revealed that the full-length *AK044422 *transcript is expressed in the mature central nervous system (Figure 2), suggesting an involvement in neurogenesis independent of *mir-124a*.

Because a large number of developmental gene-related enhancers have been shown to drive transcription in the developing forebrain during early neurogenesis [55], we examined ncRNAs expressed in GABANs that are associated with highly conserved enhancers. We identified 3 ncRNAs that were associated with highly conserved elements driving transcription in the forebrain [Additional file 10]. Two of these ncRNAs were associated with ultraconserved enhancers at the *Dlx *genomic loci, one of which, *Evf-2*, was previously shown to regulate binding of the Dlx2 transcription factor to the ultraconserved enhancer to activate the expression of the encompassed *Dlx6 *gene during neurogenesis [31].

We also identified a similar ncRNA antisense to the *Dlx1 *gene. Both *Dlx1 *and the antisense ncRNA, *Dlx1AS*, are upregulated during GABAN differentiation but downregulated during OL differentiation [Additional file 10]. These observations are consistent with published data showing that *Dlx1 *and *Dlx2 *promote GABAN lineage specification by suppressing the important oligogenic bHLH transcription factor, *Olig2 *[56] and are necessary for tangential migration of ventral forebrain-derived progenitor cells [17,56-58]. *Dlx1AS *is transcribed from an ultraconserved enhancer that drives expression in the midbrain, forebrain and olfactory system during development and in regions associated with neurogenesis in the adult mouse brain [7]. The third ncRNA, *AK005755*, which is also transcribed from an ultraconserved enhancer driving expression in the developing forebrain, has not been previously described and is not associated with any annotated developmental genes. Nonetheless, expression of this spliced transcript during GABAergic neurogenesis, coupled with its extremely conserved enhancer, suggests it plays a role in the developing forebrain.

### Specification of oligodendrocyte progenitors (OLPs)

Application of PDGF-AA to N/OPs for 4 days *in vitro *was sufficient to promote the elaboration of OLPs (Figure 1B). We confirmed by immunofluorescence microscopy that N/OPs express the corresponding receptor (PDGFRα) expression prior to and independent of the application of PDGF-AA [Additional file 11]. The developmental transition of N/OPs to OLs was associated with the upregulation of 665 mRNAs including a number of OL-selective genes, such as *Olig1 *and *Olig2*, and the downregulation of 650 mRNAs [Additional file 1]. We also found that 42 ncRNAs were downregulated, whereas 58 ncRNAs were upregulated, six of which exhibited highly correlated expression profiles to *Olig1*, having a distinct peak during OLP differentiation (Pearsons R^2 ^> 0.9) [Additional file 12; Additional file 13].

To identify ncRNAs with specific roles in OL lineage commitment, we selected ncRNAs that were upregulated in OLPs but downregulated in GABANs. This analysis identified only one ncRNA, a novel 1.2 kb transcript (*AK079380*) organized bi-directionally to the *Sox8 *gene (Figure 2). *Sox8 *is a member of the SRY-box transcription factor family, many of which play key roles in OL lineage elaboration, including *Sox9 *and *Sox11 *for OL specification and *Sox8 *and *Sox10 *for progressive OL maturation [59,60]. The ncRNA we identified, which we termed *Sox8OT (Sox8 Opposite Transcript)*, exhibited concordant expression (Pearsons R^2 ^> 0.9) to the *Sox8 *mRNA. Furthermore, a positionally conserved homologous *Sox8OT *transcript is also present in humans (*BC098409*) and rats (*BM382844*), suggesting the bi-directional relationship between *Sox8 *and *Sox8OT *is relevant. The bidirectional organization of the *Sox8 *gene and the *Sox8OT *ncRNA suggests that these transcripts are subject to common modes of regulation, possibly by sharing a promoter sequence. Indeed, recently published genome-wide maps of chromatin modifications [61] indicate a shared domain subject to dynamic modifications during the transition from embryonic stem to neuronal progenitor cells [Additional file 14]. Together, these data suggest *Sox8OT *functions in OL lineage commitment, possibly by regulating the expression of the associated *Sox8 *gene. As an alternative strategy to identify ncRNAs with specific roles in OL lineage commitment, we identified ncRNAs that were differentially expressed only in OLPs, and found that 16 were downregulated while 14 were upregulated including *Dleu2*, a previously described ncRNA overlapping but transcribed in the opposite direction of the *Trim13 (RFP2/LEU5) *gene whose expression is upregulated during early OL maturation and terminal differentiation [62].

### Progressive stages of OL lineage maturation including myelination

The withdrawal of OL cytokines (PDGF-AA) from OLP culture conditions results in cell cycle exit (Figure 1A), though the actions of environmental factors such as triiodothyronine, cyclin dependent kinase inhibitors (CDKIs: p27^KIP1^), inhibitory HLH transcription factors (ID4) and other transcription factors (YY1) that participate in the combinatorial molecular programs regulating this process [23,63-66]. In PMOs, we identified 768 upregulated mRNAs including genes involved in lipid metabolic processes (11.4%, p < 0.02), such as *Mbp*, a structural component of the myelin sheath [67]. We also observed downregulation of 562 mRNAs including genes involved in cell division (32.7%, p < 0.01), mitosis (23.4%, p < 0.01), DNA replication (13.8%, p < 0.01), and chromosome organization and biogenesis (8.65%, p < 0.01). Similarly, OLP cell cycle exit was accompanied by the upregulation of 38 ncRNAs and the downregulation of 26 ncRNAs. Many of the ncRNAs are associated with genomic loci encompassing protein-coding genes with previously described functions in development and maturation. For example, we found a highly conserved ncRNA, *AK019302*, hosted within the intron of *Bai3*, which was downregulated in PMOs. *Bai3*, which exhibits a similar expression profile, is a developmentally expressed angiostatic gene that is downregulated in the early phases of brain ischemia and in high-grade gliomas [68] and has been associated with mental retardation [69] and schizophrenia [70].

OL terminal differentiation is associated with the outgrowth of cytoplasmic processes and the accelerated production of myelin [71], and this process is regulated by neural cytokines, various classes of transcription factors, CDKIs (p21^CIP1^), and epigenetic factors [72,73]. In MYOs, we found 733 upregulated mRNAs that were enriched for genes involved in lipid metabolic processes (8.7%; p < 0.01), biopolymer metabolic processes (21.2%, p < 0.01), and actin cytoskeleton organization (8.3%, p < 0.01), including a dramatic increase in the expression of myelin genes such as *Plp1, Mbp *and *Mal*. In addition, we found 51 ncRNAs that were upregulated during OL terminal differentiation and myelination, including 8 that were broadly upregulated during progressive stages of OL lineage elaboration, exhibiting a highly correlated (Pearsons R^2 ^> 0.9) [Additional file 9; Additional file 12] expression profile with *Mog*, an important glycoprotein found on the surface of MYOs [74]. In contrast, we found that 35 ncRNAs were downregulated, including 27 ncRNAs that exhibited a highly correlated expression profile (Pearsons R^2 ^> 0.9) [Additional file 12; Additional file 13] to *Melk*, a factor important for promoting NSC proliferation [1,75] that was downregulated during OL lineage elaboration.

A variety of ncRNAs were broadly upregulated in OLPs, PMOs and MYOs including *AK141895 *and *AK133808*, associated with the *Slc44a1 *and *Rab11b *genomic loci, respectively. In these cases, both the ncRNAs and the protein-coding transcripts were strongly upregulated during OL lineage elaboration suggesting that they may function coordinately to promote appropriate OL maturation and expression of myelin proteins. We similarly identified a ncRNA antisense to *Ecsit*, an important node between BMP and Toll-Like Receptor (TLR) pathways that integrates morphogenetic signaling with pathogen recognition and innate immunity during development [76,77]. The ncRNA, *AK050588*, is upregulated in GABANs, OLPs and MYOs, in contrast to *Ecsit*, which is downregulated in these species. The observed inverse expression pattern between *AK050588 *and *Ecsit *is similar to a correlation we recently observed in embryonic stem cell differentiation [7] and suggests coordination between the expression profiles of this pair of transcripts.

### Epigenetic reprogramming of cell fate with trichostatin A (TSA)

**Figure 3:**
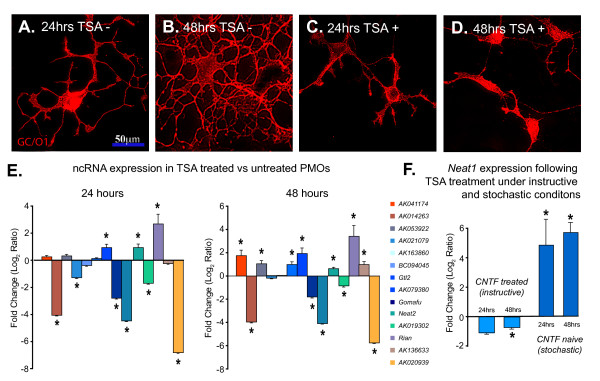
**The HDAC inhibitor, TSA prevents the acquisition of secondary morphological features of differentiating PMOs with concurrent alteration in the expression profiles of ncRNAs**. (A-D) Immunofluorescence micrographs demonstrating the profiles of OL lineage species in the absence (A, B) or presence of TSA (C, D) at 24 h and 48 h. (E) The fold change in ncRNA expression in TSA treated versus untreated PMOs at 24 h and 48 h. (F) The fold change in *Neat1 *expression in TSA treated versus untreated PMOs under CNTF presence (instructive) or absence (stochastic) with concurrent PDGF-AA factor withdrawal. Immunofluorescence micrographs of CNTF naïve, TSA treated cells and fold-change as determined by Q-PCR for remaining ncRNAs is illustrated in Additional file 15. (Error bars indicate standard error with asterisks indicated significant fold change at p > 0.05).

Histone deacetylation has been implicated in modulating OL lineage progression, particularly for developmental species undergoing cell cycle exit and progressive stages of cellular differentiation characterized by the acquisition of a branched morphology and the onset of myelin gene expression [25]. Treatment with TSA, an HDAC inhibitor, prevents progression of OLPs into PMOs with the resulting preservation of a simplified morphological phenotype that does not display secondary branching characteristic of differentiating PMOs (Figure 3). The inhibition of HDAC suppresses OL-specific gene expression and reprograms these cells into a less lineage committed state receptive to neurogenic or gliogenic differentiation signals [22-24,26,78-81]. Given that we observed a number of ncRNAs exhibiting specific expression profiles during OL lineage progression, we were interested to examine whether their expression was similarly regulated by histone deacetylation. Changes to ncRNA expression in response to HDAC inhibition would suggest the incorporation of these ncRNAs within a genetic program that contributes to progression of either neuronal or oligodendrocyte differentiation.

We employed QRT-PCR to examine the expression of ncRNAs identified within this study as strong candidates for functional roles in OL or neuronal differentiation. Broadly, we found parallel changes in cellular phenotype and alterations in ncRNA expression profiles induced by TSA. In addition to examples described above, we included other ncRNAs associated with neural developmental genes that exhibited representative developmental expression profiles. Almost all selected ncRNAs (14 of 15) exhibited significant (p < 0.05) changes in expression at 24 or 48 h (Figure 3). The majority of these changes were sustained or developed during later stages (i.e., at 48 h), suggesting the expression of these ncRNAs is subject to regulation by mechanisms of histone deacetylation.

While we observed both increased and decreased expression of long ncRNAs, the majority was downregulated, often dramatically, supporting the putative role of these ncRNAs in contributing to the progressive differentiation of OLs (Figure 3). Downregulated ncRNAs included those associated with protein-coding genes involved in progressive OL differentiation. In addition to those previously described including *Sox8OT, Gomafu*, *AK014263 *(hosted in an intron of *Csmd1*) and *AK019302 *(hosted in an intron of *Bai3*), we also observed dramatic downregulation of ncRNA *AK020939 *which is hosted within an intron of *Lhfpl3*, a member of the tetraspanin proteins which promote proliferation, migration and differentiation of OLs [82,83].

Recent evidence has shown that histone deacetylation is responsible for restricting neuronal gene expression, with the inhibition of HDACs inducing neuronal differentiation [84]. Therefore, it is of interest that a number of candidate ncRNAs exhibit an increase in expression in response to HDAC inhibition, possibly suggesting a contribution to neuronal rather than OL differentiation. For example, the previously described ncRNA *Gtl2 *was upregulated after TSA treatment. Indeed, within his study we observed upregulation of *Gtl2 *during GABAN differentiation. *Gtl2 *is expressed in restricted domains of the developing forebrain including ventral telencephalon, neocortex, hippocampus and diencephalon, suggesting roles in regional neuronal development and differentiation [85]. Finally, we found that *Neat1 *transcription exhibited complex transcriptional dynamics after TSA treatment, being strongly upregulated after TSA treatment under stochastic (probabilistic) oligodendroglial specification conditions, whereas it was downregulated after TSA treatment under the influence of instructive (inductive) signals for OL lineage elaboration (see Methods; Figure 3 and Additional file 15).

## Discussion

In this study, we utilized a custom-designed microarray platform to probe the transcription of both protein-coding and noncoding transcripts during ventral forebrain-derived NSC lineage restriction, neuronal and oligodendrocyte (OL) lineage specification, neuronal-glial fate transitions, and progressive stages of OL lineage elaboration including myelination. Expression profiles for mRNAs in our study were similar to previously reported findings [62,86] showing modulation of key signaling pathways, transcription factors, and epigenetic regulators that help to define the molecular codes governing neural lineage elaboration. Additionally, we also observed expression of components of the myelin sheath.

Although we examined protein-coding genes, we focused primarily on characterizing the expression profiles of long ncRNAs, which are being increasingly appreciated in the regulation of developmental gene expression programs [3,4,7,28]. Previous studies of neural differentiation have reported expression profiles for only a small number of long ncRNAs, such as *Nkx2.2AS *and the imprinted *H19 *ncRNA [62,86,87]. In contrast, we examined a large spectrum of long ncRNAs and found dramatic changes in expression that are unique to specific stages of cellular differentiation. Many of these ncRNAs are transcribed from complex genomic loci that encompass protein-coding genes with defined roles in neuronal and glial lineage elaboration, and these pairs of ncRNAs and associated protein-coding genes often exhibited coordinated expression during developmental transitions. Therefore, we suggest that these ncRNAs may influence the expression of the associated protein-coding genes similar to previously characterized examples such as *Nkx2.2AS *[87], *HOTAIR *[29], *p15AS *[30], *p21AS *[15] and *Evf2 *[31]. For example, many ncRNAs were associated with transcription factors that were themselves differentially regulated during OL differentiation [62,86]. Long ncRNAs, such as those identified in this study, may therefore contribute to the combinatorial transcriptional codes involved in lineage specification and terminal differentiation including myelination.

The coordinated expression of long ncRNAs with critical neural developmental protein coding genes suggests that both are subject to shared regulatory mechanisms and that long ncRNAs are integrated into complex environmentally-mediated neural and glial developmental gene expression programs. We, therefore, considered whether the well-documented ability of epigenetic mechanisms to promote decisive changes in neuronal and glial gene expression programs also includes the capacity to regulate long ncRNA expression. Indeed, treatment with the HDAC inhibitor, TSA, effectively arrests the developmental progression of OLPs into PMOs [23] and also affects the expression of long ncRNAs, suggesting that these are not only regulated by epigenetic modifications but also these transcripts are incorporated into a broader lineage specific developmental gene expression program. Furthermore, HDAC inhibition also has differential effects on certain ncRNAs in an environmentally responsive manner. For example, treatment of OLPs with TSA in the presence (inductive) or absence (stochastic) of CNTF results in opposing profiles of *Neat1 *expression, implying that long ncRNAs might be deployed in a context-specific manner to dynamically modulate seminal fate decisions within the OL lineage. Although the use of HDAC inhibitors has been essential for interrogating the epigenetic mechanisms that govern OL lineage maturation, the cellular effects of TSA administration are complex and our understanding of the mechanisms mediating ncRNA expression are still evolving. Nonetheless, long ncRNAs may represent important targets for regenerative therapies in the CNS because they exhibit both exquisite site-selective as well as genome wide effects [5,13] associated with dynamic OL developmental transition states characterized by considerable epigenetic flux [22-24,26,78,79,81].

## Conclusions

This study is the first systematic examination of the expression of long ncRNAs in forebrain-mediated NSC maturational processes, specifically OL differentiation and development. It is also the first demonstration that long ncRNAs, many of which are involved in regulating epigenetic processes, are also themselves subject to regulation by epigenetic processes. It adds to a growing consensus that not only are large portions of the mammalian genome transcribed [27] but also expressed in a regulated manner [7]. The specific expression of ncRNAs has been recently observed in other developmental programs and comparisons between these studies reveal both common and unique features [7,88]. Because the long noncoding transcriptome is still relatively poorly annotated and only partially interrogated by this study, it seems likely that many more long ncRNAs will be shown to be expressed during development and their importance for neuronal and glial biology will be more fully understood and appreciated. These studies will have particular relevance in the future as the role of noncoding transcripts in neurological disease states becomes increasingly recognized [89].

## Methods

### Neural stem cell cultures

Early embryonic (E14) multipotent and more lineage-restricted progenitor species derived from the ventral forebrain were plated at clonal density (1000 cells/mL) and propagated in serum free media (SFM) containing specified factors for various time intervals, and subsequently examined by immunofluorescence microscopy to define neural lineage profiles, expression of selected transcription factors (Figure 1) and to detect cellular proliferation (BrdU, 10 μM, added 3 h prior to fixation) as previously described [20,90-92]. Multipotent progenitor clones were generated by application of basic fibroblast growth factor (bFGF, 10 ng/mL) and were cultured 7 days in vitro (DIV) and were passaged twice to enrich the self-renewing NCSs. Subsequently, NSC clones were dissociated by trypsinization and were exposed to bFGF and the N-terminal active form of Shh (N-Shh, 50 ng/ml) for 2 DIV to generate N/OP clones [20]. GABANs were generated from N/OPs by the application of BMP2 (10 ng/mL) for 2 DIV. OLPs were generated from N/OPs by the application of PDGF AA (10 ng/mL) for 4 DIV [Additional file 11] [20,71]. PMOs and MYOs were generated from on 6 DIV late-stage OLPs by withdrawal of PDGF and concurrent addition of CNTF (20 ng/mL) for an additional 2 DIV and 4 DIV respectively [65,71]. We followed institutional IACUC guidelines for experiments in which primary mouse tissue specimens were used (Einstein Institute for Animal Studies protocol # 20080709).

### Trichostatin A (TSA) treatment

The application of TSA (10 ng/mL, Sigma) to OLs was done at the time of withdrawal of PDGF in the presence (inductive oligodendrogliogenesis) or absence (stochastic oligodendrogliogenesis) of CNTF that is necessary to promote cell cycle exit and OL differentiation. In addition to study of the four developmental stages encompassing oligodendrogliogenesis (N/OPs → OLPs → PMOs → MYOs), we also analyzed the expression patterns of selected ncRNAs at the time of PDGF withdrawal without CNTF or TSA addition (T_0_), and at 24 h and 48 h after TSA treatment in the presence and absence of CNTF [72,93]. We also compared these values to data obtained from OLs not treated with TSA at 24 h and at 48 h [25].

### Immunofluorescence microscopic analysis

This analysis was performed as previously described [72,93].

### Antibody preparations

Antibodies against the neuroepithelial marker, nestin (mIgG1, 1:200, Pharmingen) were used to label both NSCs and N/OPs. N/OPs were distinguished from NSCs by the expression of the bHLH factors, Olig2 (goat IgG, 1:500, R&D) and Mash1 (mouse, mIgG1, 1:250, Pharmingen). Progressive stages of OL lineage elaboration were documented by the expression of the OL progenitor marker, O4 (mIgM, 1:600 Sigma), the PMO marker, GC/O1 (mIgM, 1:300 Chemicon) and the MYO marker, MBP (mIgG2b, 1:500 Sternberger Monoclonals Inc.). GABANs were identified by immunoreactivity against GABA (rabbit IgG, 1:1000 Sigma). Expression of PDGFRα on N/OPs was assessed using an antibody against PDGFRα (goat IgG, 1:300, R&D Systems). Nuclear and cell cycle-associated reagents (BrdU, mIgG1, 1:400, Novocastra; Hoechst 33342, 2 μg/mL) were utilized to assess cell proliferation.

### Growth factors

To generate the various neural stem, progenitor and more lineage-restricted neuronal and OL lineage species required for immunocytochemical analysis, the following growth factor preparations were utilized: recombinant bFGF (Collaborative Biomedical Products), N-Shh, PDGF-AA and CNTF (R&D Systems) and BMP2 (gift from Genetics Institute).

### RNA preparations

RNA from mouse tissues and cell cultures was purified using Trizol (Invitrogen) or RNeasy Mini Kit (Qiagen) and treated with DNase I (Invitrogen), according to the protocols provided by the manufacturers. The quality of purified total RNA samples was assessed with an RNA 6000 Nano assay kit using the Agilent 2100 Bioanalyzer (Agilent Technologies) according to the manufacturer's instructions. For microarray experiments, RNA was amplified and labeled using the Amino Allyl Message Amp II kit (Ambion) following the instructions provided by the manufacturer. Amplified aRNA from each time point, as well as a reference pooled sample comprising a mixture of RNA from all time points, was labeled with either Cy3 or Cy5 monoreactive dyes (Amersham Biosciences) according to the MessageAmp II protocol (Ambion). The quality and quantity of amplified RNA samples were assessed using the Agilent 2100 Bioanalyzer as described above.

### Microarray expression profiling

The microarrays contained 22,038 65-mer oligonucleotide probes from the Mouse OligoLibrary (Compugen, San Jose, CA, USA) and 2,118 70-mer oligonucleotide probes that were designed to target ncRNAs, including known mouse pre-miRNAs from miRBase [94], longer mouse ncRNAs from RNAdb [1], and "high confidence" ncRNAs identified from the FANTOM3 project [1]. The custom 70-mer probes were printed alongside Mouse OligoLibrary probes on Power Matrix slides (Full Moon BioSystems, Sunnyvale, CA, USA) at the SRC Microarray Facility (University of Queensland, Brisbane, Australia). The quality of the print run was verified by hybridizing random 10-mer oligonucleotides to the 1^st ^and last slides of the run. The array design is available from the ArrayExpress Data Warehouse (EMBL-EBI; ArrayExpress Accession Number A-MEXP-1070).

Labeled RNAs from each of the five developmental stages was hybridized with the NSC sample to individual microarrays. Three biological replicates were performed for each time point. Blocking, hybridization and washing was performed according to the manufacturer's instructions (Full Moon BioSystems, Sunnyvale, CA, USA). Slides were scanned at 5 μm resolution using a DNA microarray scanner (Agilent Technologies). Feature extraction was performed using ImaGene software (BioDiscovery, El Segundo, CA, USA), with manual grid adjustment and auto-spot finding and segmentation. Data was exported from ImaGene as text files, then uploaded and analyzed using the Linear Models for Microarray Data (LIMMA) software package via the R Project for Statistical Computing http://www.r-project.org. Data was background-corrected, normalized both within and between arrays [95], and differential expression analysis was performed by fitting a linear model of the data to the experimental design matrix and then calculating Bayesian statistics (B statistics; posterior log odds) adjusted for multiple testing using Benjamini-Hochberg analysis [96]. Raw and processed microarray data is available at the ArrayExpress Data Warehouse (EMBL-EBI; ArrayExpress Accession Number E-TABM-874).

### PCR primers

The primers were designed and synthesized (Invitrogen) to amplify selected ncRNA sequences [Additional file 16].

### Quantitative real-time PCR

Quantitative real-time PCR (QRT-PCR) was performed according to manufacturer's protocols (Qiagen). QRT-PCR reactions were performed in a 96-well plate, each reaction consisted of a 25 μL mixture including 12.5 μL of 2× QuantiFast SYBR Green PCR Master Mix (Qiagen), 1 μM of forward and reverse sequences of the corresponding primers and 40 ng of cDNA. Mouse 18S ribosomal RNA was used as an internal control to ensure accuracy of the results. QRT-PCR amplifications were performed using the ABI PRISM 7000 Sequence Detection System (Applied Biosystems). Amplification conditions were as follows: 5 min at 95°C to activate the HotStarTaq *Plus *DNA Polymerase followed by 40 cycles of 10 s at 95°C and 30 s 60°C. QRT-PCR conditions were optimized by adjusting the primer concentrations and the quantity of cDNA. A dissociation curve was performed to ensure the validity of each specific PCR product. QRT-PCR results were analyzed using Relative Expression Software Tool [97] software http://rest.gene-quantification.info/.

### Classification of probes targeting protein-coding or non-protein-coding transcripts

Although the Mouse OligoLibrary probe set was predominantly designed to recognize known or putative protein-coding transcripts, several thousand probes targeted miscellaneous cDNAs and ESTs whose coding status was not well characterized at the time this commercial probe set was first produced. To update the annotation of these probes and to clarify whether they targeted protein-coding or noncoding regions, a computational pipeline was designed to re-annotate the entire probe set. Sequences for all probes were mapped to the February 2006 (NCBI Build 36) assembly of the mouse genome using BLAT [98] (parameters: minScore = 50, minIdentity = 99, stepSize = 5, tileSize = 11, ooc = 11.ooc). Probes that could not be reliably mapped were excluded from the study. Targeted transcripts were then defined as protein-coding and noncoding as described previously [7]. The genomic context of ncRNAs (relative to protein-coding genes) was also determined as described previously [6].

### Conservation and secondary structure predictions of ncRNAs

The secondary structural composition of expressed ncRNAs was determined by intersecting their chromosomal positions with those of the RNAz structural predictions made across the entire mouse genome (using confidence threshold levels of P > 0.5 and P > 0.9), which were performed as previously described [6,34]. High confidence predicted RNA secondary structures were rendered with CONTRAfold [99].

### Gene Ontology (GO) term and microRNA target enrichment analysis

The Gene Ontology project http://www.geneontology.org/ provides a controlled vocabulary to functionally annotate genes. By studying GO terms associated with a large set of genes, one obtains useful information about the types of genes represented. Lists of differentially expressed protein-coding genes were uploaded into FastiGO+ http://babelomics.bioinfo.cipf.es[100]. Statistically over-represented GO terms in the biological process and molecular function and microRNA target enrichments were obtained by applying a p-value cutoff as indicated, correcting for multiple testing with Benjamini False Discovery Rate.

## Authors' contributions

TRM, MED and IAQ analyzed the microarray data and drafted the manuscript. SG participated in the study design, carried out cell culture studies, immunohistochemical protocols and helped to draft the manuscript. GL performed microarray studies and additional molecular genetic assays. JSM and MFM conceived the study, participated in its design and coordination, and helped to draft the manuscript.

## Supplementary Material

Additional file 1 **Expression of mRNAs according to microarray analysis at different stages of oligodendrocyte and neuronal differentiation**. Abbreviations are: bipotent progenitor cells, N/OP; GABAergic neurons, GABAN; oligodendrocyte progenitors, OLP; post-mitotic oligodendrocytes, PMO; myelinating oligodendrocytes: MYO). Probe IDs for targeted mRNA may be queried in the NRED database [102]. Information for targeted mRNAs shown includes Accession IDs, UniGene Common Name and Symbol and M-statistic, Fold Change, A- and B-statistic as calculated from microarray analysis. Click here for file

Additional file 2 **Expression of ncRNAs according to microarray analysis at different stages of oligodendrocyte and neuronal differentiation**. Abbreviations are: bipotent progenitor cells, N/OP; GABAergic neurons, GABAN; oligodendrocyte progenitors, OLP; post-mitotic oligodendrocytes, PMO; myelinating oligodendrocytes: MYO). Probe IDs for targeted mRNA may be queried in the NRED database [102]. Information for targeted ncRNAs shown includes Accession IDs, M-statistic, Fold Change, A- and B-statistic as calculated from microarray analysis. Click here for file

Additional file 3 **QRT-PCR validation for microarray analysis of ncRNA expression in OL lineage elaboration**. QRT-PCR analysis was performed to evaluate expression of 11 ncRNA across five cell types and corroborated microarray data in 48 (87%) of 55 instances. Click here for file

Additional file 4 **Noncoding RNAs associated with protein-coding genes**. Noncoding transcript accession IDs are shown with associated protein-coding UniGene Common Name and Symbol and relationship of association (intronic, bidirectional, antisense). Evidence for positional conservation of ncRNA with homologous gene in the human genome (hg18) indicated. Click here for file

Additional file 5 **Noncoding RNA transcripts sequences predicted to fold into high confidence (P > 0.9) secondary structures according to RNAz**. Chromosome coordinates and sequences are provided for region of expressed ncRNAs that are predicted to fold into conserved secondary structures. Predicted secondary structures according are provided in dot-bracket annotation (analysis conducted with Vienna RNA Package [101]). Selected high confidence structures are illustrated in Additional file 6. Click here for file

Additional file 6 **Rendered illustrations of the top five most stable secondary predicted structures in expressed ncRNAs**. The five most predicted stable structures were rendered using CONTRAfold [99]. Click here for file

Additional file 7 ***Gomafu *expression during GABAergic neuronal and progressive stages of OL lineage elaboration**. (A) Relative expression of *Gomafu *during GABAN and OL differentiation (expression is relative to NSCs and error bars show standard deviation). *Gomafu *is exclusively downregulated in N/OPs, but upregulated in all other sampled cell stages. (B) *In situ *hybridization of sagittal adult mouse brain sections for *Gomafu *expression. Whole brain is shown in top left panel, hippocampus top right panel, coronal section of olfactory bulb in bottom left panel, and sagittal section of olfactory bulb and cortex in bottom right panel. *Gomafu *is expressed in the cortex (green arrow), hippocampus and mitral layer of the olfactory bulb (red arrow). *Gomafu *does not exhibit expression in the cerebellum (blue arrow). *Images courtesy of the Allen Brain Atlas *http://www.brain-map.org. Click here for file

Additional file 8 ***Snhg10 *exhibits specific expression profiles in the adult mouse brain**. *Snhg10 *exhibits a strong and broad expression throughout the whole mouse brain (A), with specific expression in Purkinje cells in the cerebellum (B) and hippocampus (C). *Images courtesy of the Allen Brain Atlas *http://www.brain-map.org. Click here for file

Additional file 9 **mRNAs and ncRNAs that exhibit discordant expression trends during neuronal-glia fate switching**. Table shows probe IDs that may be queried in the NRED database (Dinger et al., 2008), UniGene Common Name and Accession IDs and the discordant M-statistic and fold change associated with neural stem cell to GABAergic neuron (GABAN vs NSC) or oligodendrocyte progenitor (OLP vs NSC) transition as determined by microarray analysis. Click here for file

Additional file 10 **Expression of ncRNAs associated with ultraconserved elements**. (A) Genomic context of the *Dlx1 *and *Dlx2 *gene (dark blue), the ncRNA *Dlx1AS *(AK132348; red) showing the position of ultraconserved element with previously described enhancer function (VISTA 422; green) and histogram of vertebrate conservation (dark blue). (B) Enhancer (VISTA 422) function driving reporter gene expression in the developing forebrain (red arrow) of 11.5 day mouse embryo [55]. *Images courtesy of VISTA Enhancer Browser *http://enhancer.lbl.gov/frnt_page.shtml. (C) Expression of *Dlx1AS *(red) and *Dlx1 *gene (blue) during OL differentiation (expression is relative to NSCs and error bars show standard deviation). *Dlx1AS *ncRNA is upregulated in GABAN, similar to *Dlx1*, but downregulated in N/OPs and in different stages of OL differentiation (OLPs, PMOs, MYOs). (D) Genomic context of the *Dlx5 *and *Dlx6 *genes (blue) and the ncRNA *Evf *transcripts (1 and 2; red) showing the position of two ultraconserved elements with previously described enhancer function (VISTA 298) [55] and the enhancer described by Feng et al. [31]; green) and histogram of vertebrate conservation (dark blue). (E) Enhancer (VISTA 298) function driving reporter gene expression in the developing forebrain (red arrow) of 11.5 day mouse embryo [55]. *Images courtesy of VISTA Enhancer Browser *http://enhancer.lbl.gov/frnt_page.shtml. (F) Expression of *Evf *(red) and *Dlx5 *gene (blue) during oligodendrogliogenesis (expression is relative to NSCs and error bars show standard deviation). The *Evf *ncRNA (red) is upregulated during GABAN, similar to *Dlx5 *(blue), but downregulated in N/OPs and later stages of oligodendrogliogenesis (OLPs, PMOs, MYOs). (G) Genomic context of the novel *AK005755 *ncRNA (red) showing the position of; ultraconserved element with previously described enhancer function (VISTA 433; green) and histogram of vertebrate conservation (dark blue). (H) Enhancer (VISTA 433) function driving reporter gene expression in the developing forebrain (red arrow) of 11.5 day mouse embryo [55]. *Images courtesy of VISTA Enhancer Browser *http://enhancer.lbl.gov/frnt_page.shtml. Click here for file

Additional file 11 **Elaboration of PDGFRα on bipotent neuronal/oligodendrocyte precursors (N/OPs) independent of PDGF-AA application following propagation *in vitro***. N/OPs at 2 h (A-B) in vitro express the bHLH transcription factors, Olig2 and Mash1 (A), in addition to nestin (B). Immunofluorescence microscopic analysis reveals that PDGFRα is not initially expressed by this cellular species in our clonal culture paradigm. However, PDGFRα expression is unequivocally present at 24 h (C-D, arrowheads), demonstrating that N/OPs begin to acquire PDGFRα expression and responsiveness to PDGF-AA, which is required for proliferation and migration of OL progenitors following specification. Click here for file

Additional file 12 **Gene expression profiles of ncRNAs during oligodendrocyte differentiation**. Correlation of expression profiles of ncRNAs with protein-coding gene markers during oligodendrogliogenesis. Genes with well-characterized roles in oligodendrogenesis were used to identify ncRNAs with correlated expression profiles (Pearson's coefficient > 0.9). This included *Olig1 *(**A**; purple) and *Stm2 *(**B**; red) that are differentially expressed in OLPs or *Mobp *(**C**; blue) or *Melk *(**D**; pink) that are differentially expressed during terminal differentiation to MYOs. NcRNA accession IDs are available in Additional file 13. Click here for file

Additional file 13 **Noncoding RNAs that exhibit concordant expression profiles with marker genes with well-characterized roles in neuronal and oligodendrocyte differentiation**. Genes with well-characterized roles in oligodendrogliogenesis were used to identify ncRNAs with correlated expression profiles (Pearson's coefficient > 0.9). These genes include *Olig1, Stmn2, Mobp, Melk, MOG, Mash1 *and *Nkx2-2*. Illustration of selected expression profiles is shown in Additional file 13. Click here for file

Additional file 14 **The *Sox8 *gene and associated *Sox8OT *ncRNA share a dynamically modified chromatin domain**. The genome browser view shows the bidirectional organization of the Sox8 gene (blue) and the Sox8OT ncRNA (red). A shared promoter region (shaded box) exhibits dynamic chromatin remodeling in embryonic stem cells (A; ES), embryonic fibroblasts (B; MEF) and neural progenitors (C; NP) [61]. The combination of active H3K4me3 (green histogram) and repressive H3K27me3 (red histogram) modified chromatin, observed in ES and MEF cells, is termed a bivalent domain and is indicative of a gene in a 'poised' state for activation. This is reflected in the low prevalence of H3K36me3 modified chromatin (blue histogram) that is normally associated with RNA Polymerase elongation. In NPs the bivalent domain has been resolved to an active H3K4me3 domain and the presence of H3K36me3 modified domains implies that both the *Sox8 *and *Sox8OT *transcripts are concordantly upregulated. Tpm; tags per million. Click here for file

Additional file 15 **The HDAC inhibitor TSA prevents the acquisition of secondary morphological features of differentiating PMOs with concurrent alteration in the expression profiles of ncRNAs under instructive conditions (CNTF treatment) and stochastic (CNTF naïve) for OL lineage maturation**. (A-D) Immunofluorescence micrographs demonstrating the profiles of OL lineage species in the absence (A, B) or presence (C, D) of TSA at T1 (24 h, A, C) and T2 (48 h, B, D) in the presence of CNTF with concurrent PDGF-AA factor withdrawal. (E, F) The effects of TSA application on comparative expression profiles of ncRNAs as assessed at T1 (E) and at T2 (F) in relation to the timing of TSA exposure in the experimental conditions. (G-K) Immunofluorescence micrographs demonstrating the profiles of OL lineage species in the absence (G, H) or presence (J, K) of TSA at T1 (24 h, G, J) and T2 (48 h, H, K) in the presence PDGF-AA factor withdrawal only. (L, M) The effects of TSA application on comparative expression profiles of ncRNAs assessed at T1 (L) and at T2 (M) in relation to the timing of TSA exposure in the experimental conditions. Click here for file

Additional file 16 **List of ncRNA primers used in the validation of microarray results by QRT-PCR**. Click here for file
