# Supplementary material for: Long noncoding RNAs in neuronal-glial fate specification and oligodendrocyte lineage maturation
Source: BMC Neurosci. 2010 Feb 5;11:14. doi: 10.1186/1471-2202-11-14 (PMC2829031; doi:10.1186/1471-2202-11-14)
Supplement: Additional file 6 — Rendered illustrations of the top five most stable secondary predicted structures in expressed ncRNAs. The five most predicted stable structures were rendered using CONTRAfold [99]. [file 1471-2202-11-14-S6.PDF]

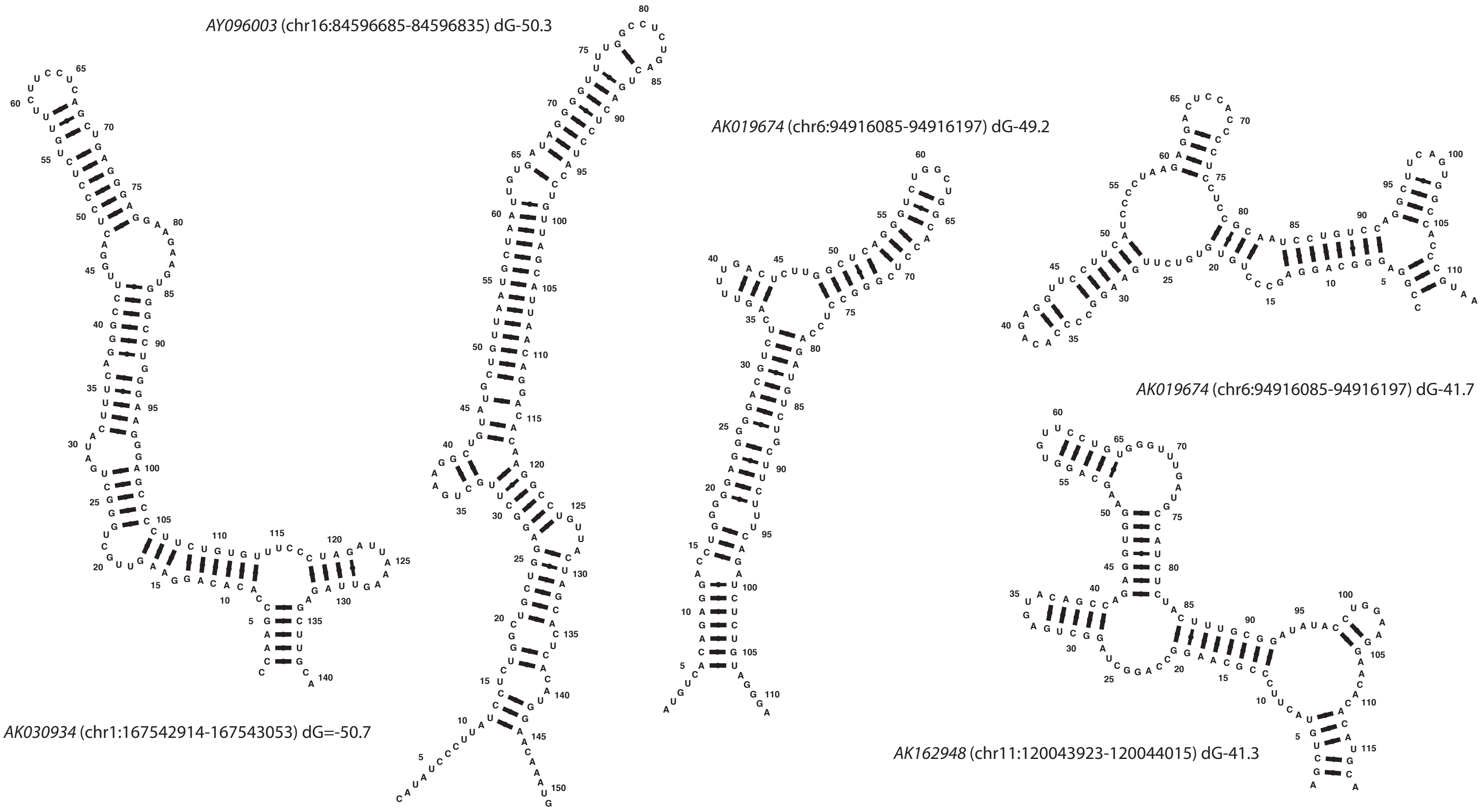


**Additional File 6. Rendered illustrations of the top five most stable secondary predicted structures in expressed ncRNAs.** The five most predicted stable structures were rendered using CONTRAfold [99].
