# Supplementary material for: Long noncoding RNAs in neuronal-glial fate specification and oligodendrocyte lineage maturation
Source: BMC Neurosci. 2010 Feb 5;11:14. doi: 10.1186/1471-2202-11-14 (PMC2829031; doi:10.1186/1471-2202-11-14)
Supplement: Additional file 7 — Gomafu expression during GABAergic neuronal and progressive stages of OL lineage elaboration. (A) Relative expression of Gomafu during GABAN and OL differentiation (expression is relative to NSCs and error bars show standard deviation). Gomafu is exclusively downregulated in N/OPs, but upregulated in all other sampled cell stages. (B) In situ hybridization of sagittal adult mouse brain sections for Gomafu expression. Whole brain is shown in top left panel, hippocampus top right panel, coronal section of olfactory bulb in bottom left panel, and sagittal section of olfactory bulb and cortex in bottom right panel. Gomafu is expressed in the cortex (green arrow), hippocampus and mitral layer of the olfactory bulb (red arrow). Gomafu does not exhibit expression in the cerebellum (blue arrow). Images courtesy of the Allen Brain Atlas http://www.brain-map.org. [file 1471-2202-11-14-S7.PDF]

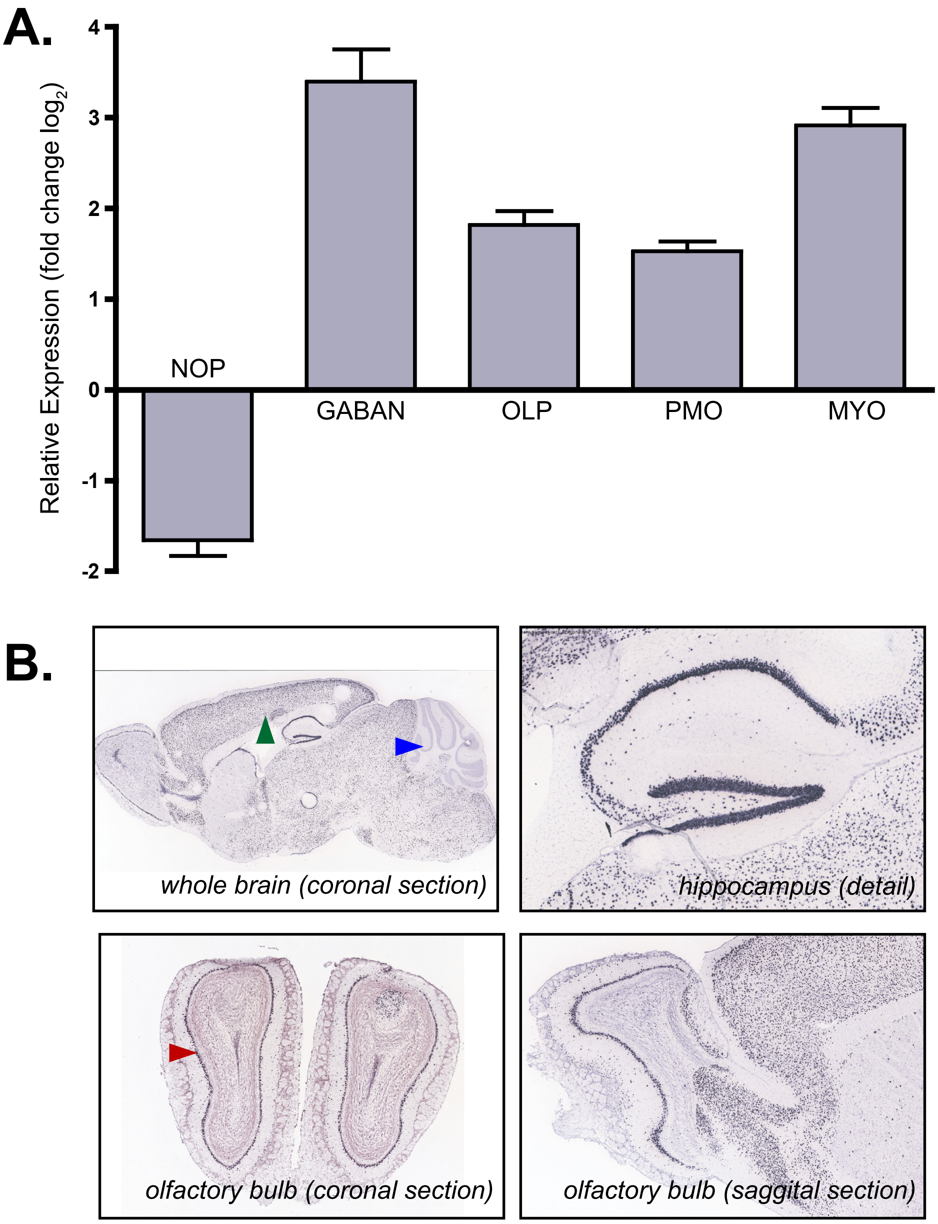

Additional File 7. *Gomafu* expression during GABAergic neuronal and progressive stages of OL lineage elaboration. (A) Relative expression of *Gomafu* during GABAN and OL differentiation (expression is relative to NSCs and error bars show standard deviation). *Gomafu* is exclusively downregulated in N/OPs, but upregulated in all other sampled cell stages. **(B)** *In situ* hybridization of sagittal adult mouse brain sections for *Gomafu* expression. Whole brain is shown in top left panel, hippocampus top right panel, coronal section of olfactory bulb in bottom left panel and sagittal section of olfactory bulb and cortex in bottom right panel. *Gomafu* is expressed in the cortex (green arrow), hippocampus and mitral layer of the olfactory bulb (red arrow). *Gomafu* does not exhibit expression in the cerebellum (blue arrow). Images courtesy of the Allen Brain Atlas (www.brain-map.org).
