# Supplementary material for: Long noncoding RNAs in neuronal-glial fate specification and oligodendrocyte lineage maturation
Source: BMC Neurosci. 2010 Feb 5;11:14. doi: 10.1186/1471-2202-11-14 (PMC2829031; doi:10.1186/1471-2202-11-14)
Supplement: Additional file 8 — Snhg10 exhibits specific expression profiles in the adult mouse brain. Snhg10 exhibits a strong and broad expression throughout the whole mouse brain (A), with specific expression in Purkinje cells in the cerebellum (B) and hippocampus (C). Images courtesy of the Allen Brain Atlas http://www.brain-map.org. [file 1471-2202-11-14-S8.PDF]

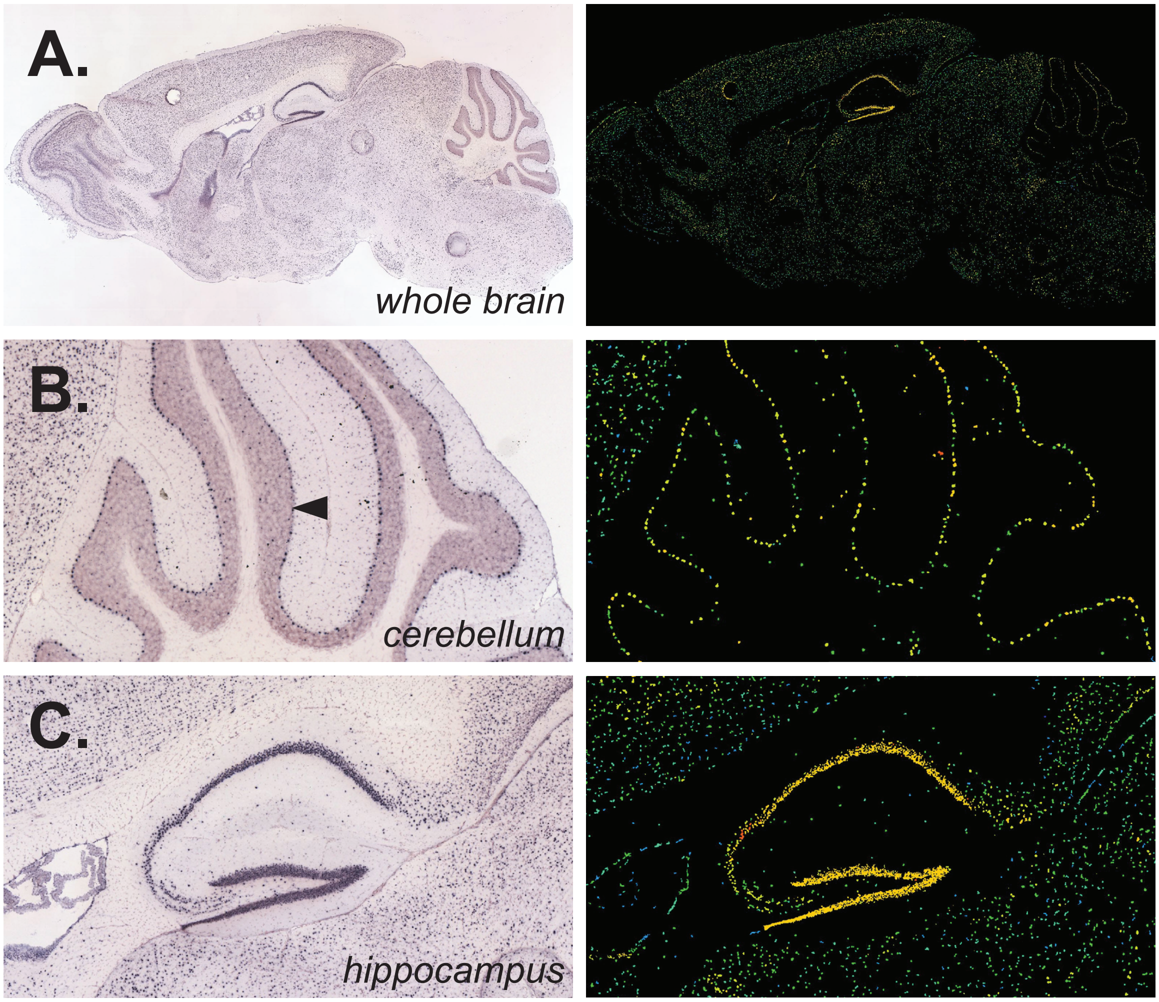

Additional File 8. ***Snhg10*** **exhibits specific expression profiles in the adult mouse brain.** *Snhg10* exhibits a strong and broad expression throughout the whole mouse brain **(A),** with specific expression in Purkinje cells in the cerebellum **(B)** and hippocampus **(C).** Images courtesy of the Allen Brain Atlas (www.brain-map.org).
