# Supplementary material for: Long noncoding RNAs in neuronal-glial fate specification and oligodendrocyte lineage maturation
Source: BMC Neurosci. 2010 Feb 5;11:14. doi: 10.1186/1471-2202-11-14 (PMC2829031; doi:10.1186/1471-2202-11-14)
Supplement: Additional file 10 — Expression of ncRNAs associated with ultraconserved elements. (A) Genomic context of the Dlx1 and Dlx2 gene (dark blue), the ncRNA Dlx1AS (AK132348; red) showing the position of ultraconserved element with previously described enhancer function (VISTA 422; green) and histogram of vertebrate conservation (dark blue). (B) Enhancer (VISTA 422) function driving reporter gene expression in the developing forebrain (red arrow) of 11.5 day mouse embryo [55]. Images courtesy of VISTA Enhancer Browser http://enhancer.lbl.gov/frnt_page.shtml. (C) Expression of Dlx1AS (red) and Dlx1 gene (blue) during OL differentiation (expression is relative to NSCs and error bars show standard deviation). Dlx1AS ncRNA is upregulated in GABAN, similar to Dlx1, but downregulated in N/OPs and in different stages of OL differentiation (OLPs, PMOs, MYOs). (D) Genomic context of the Dlx5 and Dlx6 genes (blue) and the ncRNA Evf transcripts (1 and 2; red) showing the position of two ultraconserved elements with previously described enhancer function (VISTA 298) [55] and the enhancer described by Feng et al. [31]; green) and histogram of vertebrate conservation (dark blue). (E) Enhancer (VISTA 298) function driving reporter gene expression in the developing forebrain (red arrow) of 11.5 day mouse embryo [55]. Images courtesy of VISTA Enhancer Browser http://enhancer.lbl.gov/frnt_page.shtml. (F) Expression of Evf (red) and Dlx5 gene (blue) during oligodendrogliogenesis (expression is relative to NSCs and error bars show standard deviation). The Evf ncRNA (red) is upregulated during GABAN, similar to Dlx5 (blue), but downregulated in N/OPs and later stages of oligodendrogliogenesis (OLPs, PMOs, MYOs). (G) Genomic context of the novel AK005755 ncRNA (red) showing the position of; ultraconserved element with previously described enhancer function (VISTA 433; green) and histogram of vertebrate conservation (dark blue). (H) Enhancer (VISTA 433) function driving reporter gene expression in the developing f [file 1471-2202-11-14-S10.PDF]

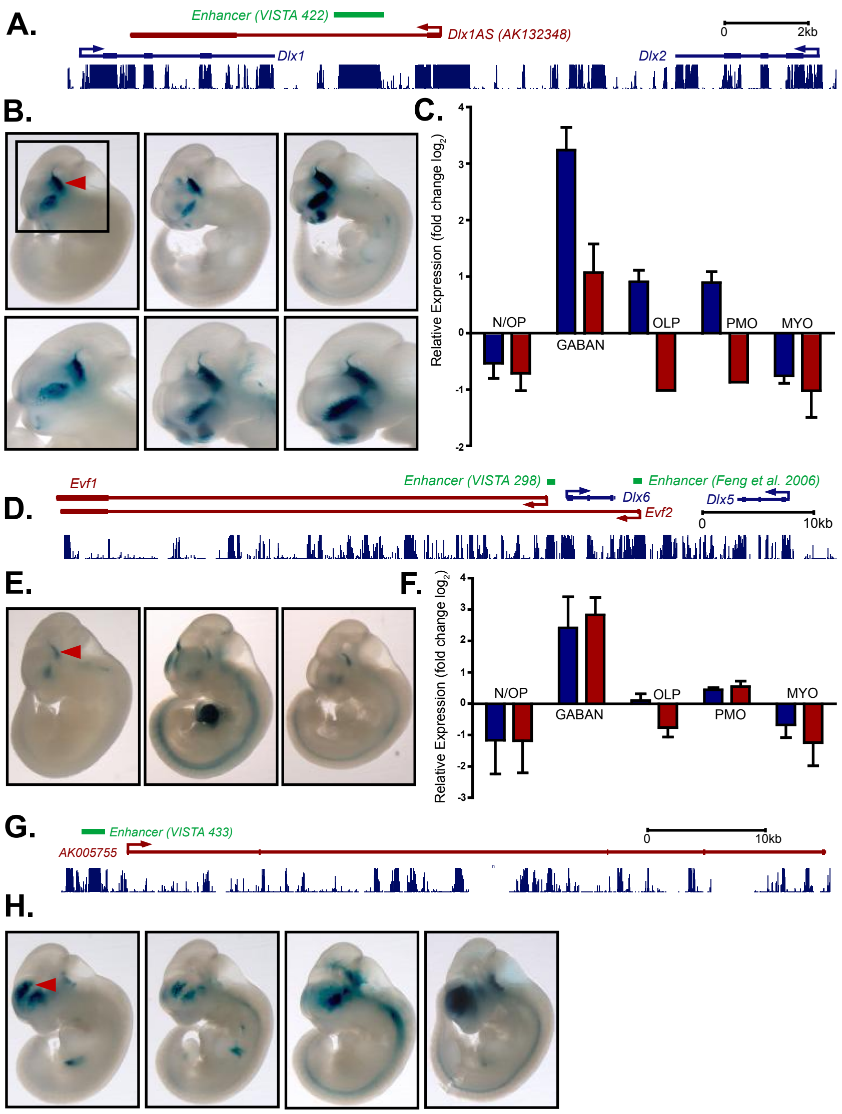

Additional File 10. Expression of ncRNAs associated with ultraconserved elements. (A) Genomic context of the *Dlx1* and *Dlx2* gene (dark blue), the ncRNA *Dlx1AS* (*AK132348*; red) showing the position of ultraconserved element with previously described enhancer function (VISTA 422; green) and histogram of vertebrate conservation (dark blue). **(B)** Enhancer (VISTA 422) function driving reporter gene expression in the developing forebrain (red arrow) of 11.5 day mouse embryo [55]. Images courtesy of VISTA Enhancer Browser (http://enhancer.lbl.gov/frnt_page.shtml). **(C)** Expression of *Dlx1AS* (red) and *Dlx1* gene (blue) during OL differentiation (expression is relative to NSCs and error bars show standard deviation). *Dlx1AS* ncRNA is upregulated in GABAN, similar to *Dlx1*, but downregulated in N/OPs and in different stages of OL differentiation (OLPs, PMOs, MYOs). **(D)** Genomic context of the *Dlx5* and *Dlx6* genes (blue) and the ncRNA *Evf* transcripts (1 and 2; red) showing the position of two ultraconserved elements with previously described enhancer function (VISTA 298 [55] and the enhancer described by Feng et al. [31]; green) and histogram of vertebrate conservation (dark blue). **(E)** Enhancer (VISTA 298) function driving reporter gene expression in the developing forebrain (red arrow) of 11.5 day mouse embryo [55]. Images courtesy of VISTA Enhancer Browser (http://enhancer.lbl.gov/frnt_page.shtml). **(F)** Expression of Evf (red) and *Dlx5* gene (blue) during oligodendrogliogenesis (expression is relative to NSCs and error bars show standard deviation). The *Evf* ncRNA (red) is upregulated during GABAN, similar to *Dlx5* (blue), but downregulated in N/OPs and later stages of oligodendrogliogenesis (OLPs, PMOs, MYOs). **(G)** Genomic context of the novel *AK005755* ncRNA (red) showing the position of; ultraconserved element with previously described enhancer function (VISTA 433; green) and histogram of vertebrate conservation (dark blue). **(H)** Enhancer (VISTA 433) function driving reporter gene expression in the developing forebrain (red arrow) of 11.5 day mouse embryo [55]. Images courtesy of VISTA Enhancer Browser (http://enhancer.lbl.gov/frnt_page.shtml).
