# Supplementary material for: Long noncoding RNAs in neuronal-glial fate specification and oligodendrocyte lineage maturation
Source: BMC Neurosci. 2010 Feb 5;11:14. doi: 10.1186/1471-2202-11-14 (PMC2829031; doi:10.1186/1471-2202-11-14)
Supplement: Additional file 11 — Elaboration of PDGFRα on bipotent neuronal/oligodendrocyte precursors (N/OPs) independent of PDGF-AA application following propagation in vitro. N/OPs at 2 h (A-B) in vitro express the bHLH transcription factors, Olig2 and Mash1 (A), in addition to nestin (B). Immunofluorescence microscopic analysis reveals that PDGFRα is not initially expressed by this cellular species in our clonal culture paradigm. However, PDGFRα expression is unequivocally present at 24 h (C-D, arrowheads), demonstrating that N/OPs begin to acquire PDGFRα expression and responsiveness to PDGF-AA, which is required for proliferation and migration of OL progenitors following specification. [file 1471-2202-11-14-S11.PDF]

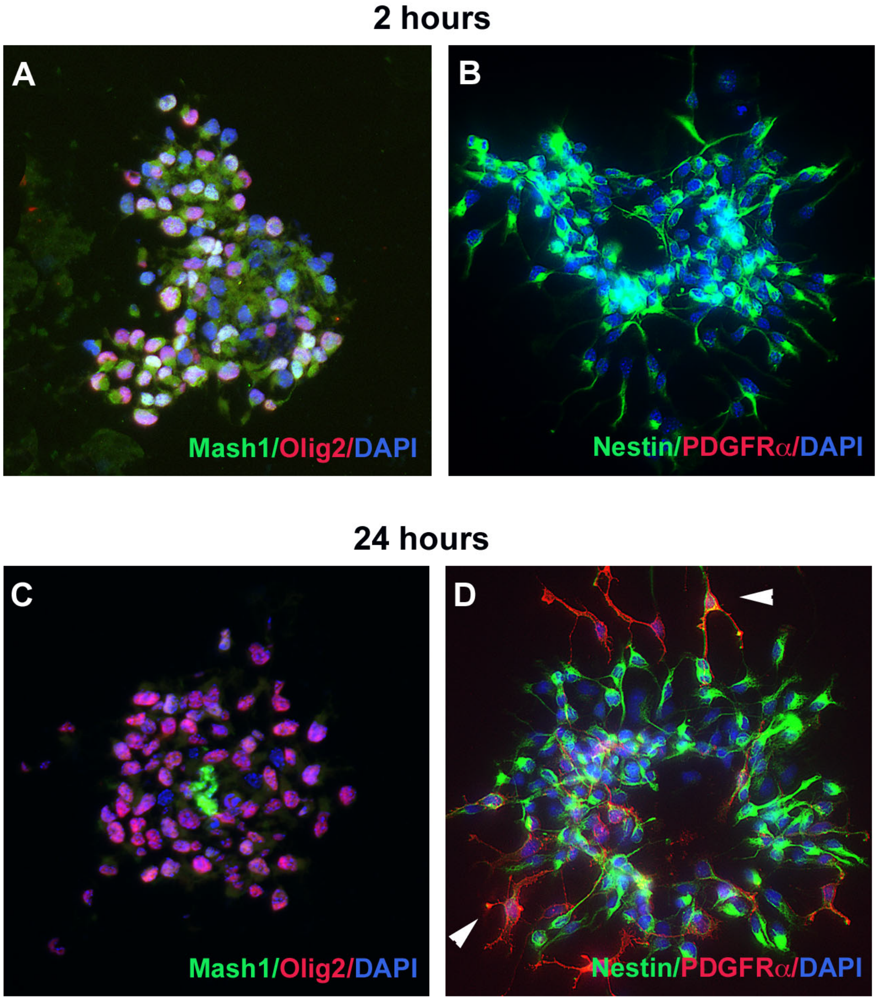

Additional File 11. Elaboration of PDGFRα on bipotent neuronal / oligodendrocyte precursors (N/OPs) independent of PDGF-AA application following propagation *in vitro.* N/OPs at 2 h. **(A-B)** in vitro express the bHLH transcription factors, Olig2 and Mash1 **(A),** in addition to nestin **(B).** Immunofluorescence microscopic analysis reveals that PDGFRα expression is not initially expressed by this cellular species in our clonal culture paradigm. However, PDGFRα (arrow heads) expression is unequivocally present at 24 h. **(C-D)** showing that N/OPs begin to acquire PDGFRα expression and responsiveness to PDGF-AA required for OL lineage commitment.
