# Supplementary material for: Long noncoding RNAs in neuronal-glial fate specification and oligodendrocyte lineage maturation
Source: BMC Neurosci. 2010 Feb 5;11:14. doi: 10.1186/1471-2202-11-14 (PMC2829031; doi:10.1186/1471-2202-11-14)
Supplement: Additional file 12 — Gene expression profiles of ncRNAs during oligodendrocyte differentiation. Correlation of expression profiles of ncRNAs with protein-coding gene markers during oligodendrogliogenesis. Genes with well-characterized roles in oligodendrogenesis were used to identify ncRNAs with correlated expression profiles (Pearson's coefficient > 0.9). This included Olig1 (A; purple) and Stm2 (B; red) that are differentially expressed in OLPs or Mobp (C; blue) or Melk (D; pink) that are differentially expressed during terminal differentiation to MYOs. NcRNA accession IDs are available in Additional file 13. [file 1471-2202-11-14-S12.PDF]

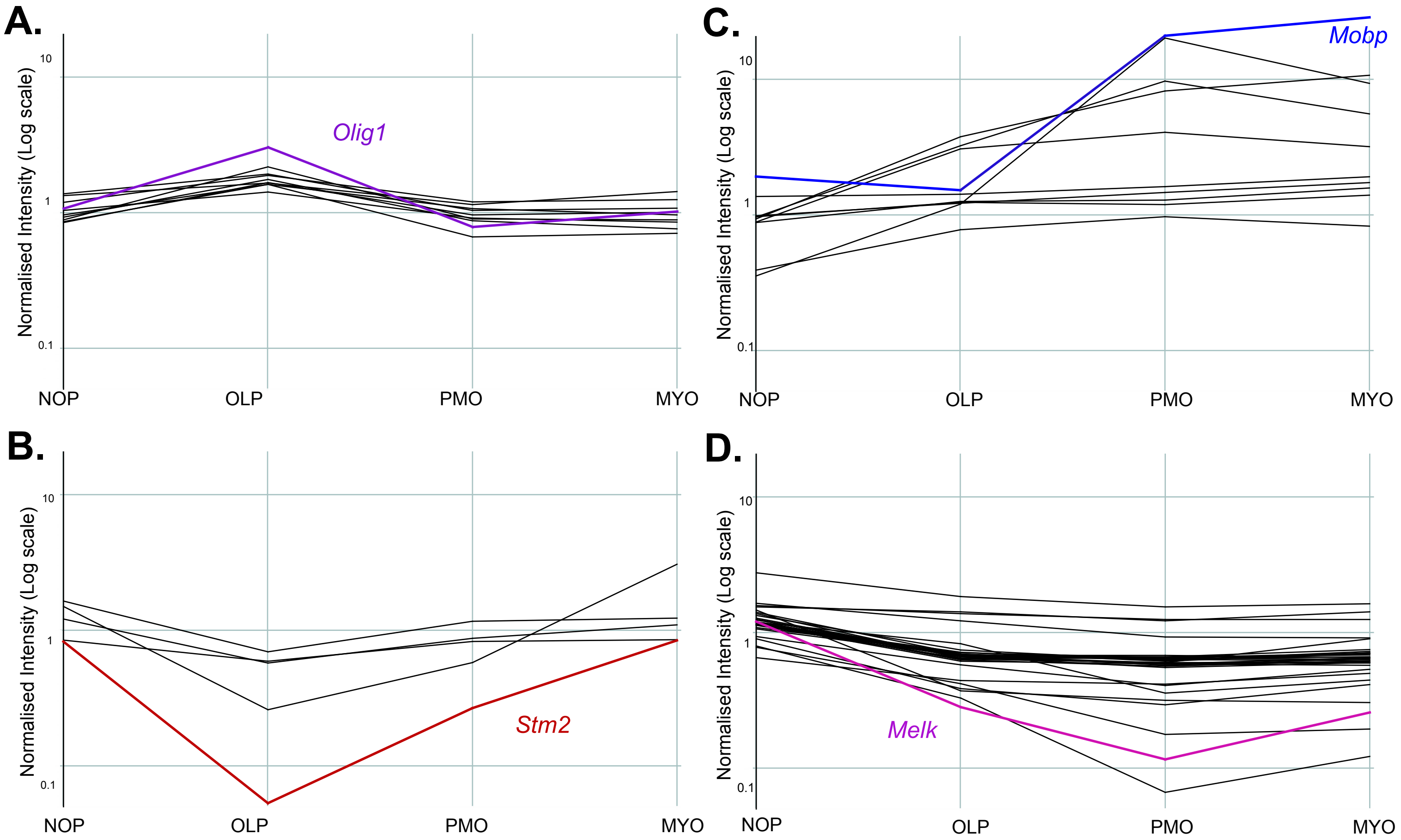

Additional File 12. Gene expression profiles of ncRNAs during oligodendroctye differentiation. Correlation of expression profiles of ncRNAs with protein-coding gene markers duringoligodendrogliogenesis. Genes with well-characterized roles in oligodendrogenesis were used to identify ncRNAs with correlated expression profiles (Pearson's coefficient > 0.9). This included *Olig1* (**A**; purple) and *Stm2* (**B**; red) that are differentially expressed in OLPs or *Mobp* (**C**; blue) or *Melk* (**D**; pink) that are differentially expressed during terminal differentiation to MYOs. NcRNA accession IDs are available in Additional File 13.
