# Supplementary material for: Long noncoding RNAs in neuronal-glial fate specification and oligodendrocyte lineage maturation
Source: BMC Neurosci. 2010 Feb 5;11:14. doi: 10.1186/1471-2202-11-14 (PMC2829031; doi:10.1186/1471-2202-11-14)
Supplement: Additional file 14 — The Sox8 gene and associated Sox8OT ncRNA share a dynamically modified chromatin domain. The genome browser view shows the bidirectional organization of the Sox8 gene (blue) and the Sox8OT ncRNA (red). A shared promoter region (shaded box) exhibits dynamic chromatin remodeling in embryonic stem cells (A; ES), embryonic fibroblasts (B; MEF) and neural progenitors (C; NP) [61]. The combination of active H3K4me3 (green histogram) and repressive H3K27me3 (red histogram) modified chromatin, observed in ES and MEF cells, is termed a bivalent domain and is indicative of a gene in a 'poised' state for activation. This is reflected in the low prevalence of H3K36me3 modified chromatin (blue histogram) that is normally associated with RNA Polymerase elongation. In NPs the bivalent domain has been resolved to an active H3K4me3 domain and the presence of H3K36me3 modified domains implies that both the Sox8 and Sox8OT transcripts are concordantly upregulated. Tpm; tags per million. [file 1471-2202-11-14-S14.PDF]

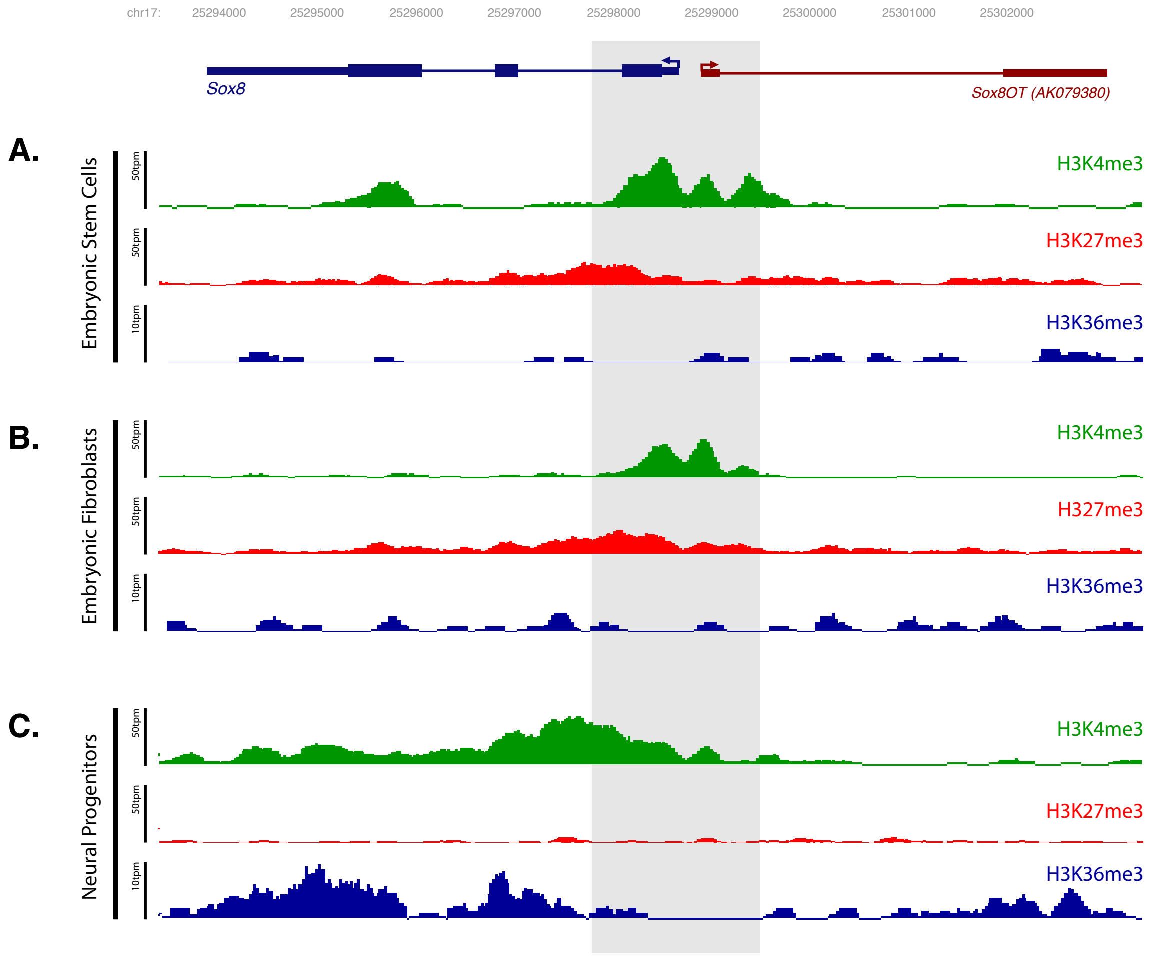

Additional File 14. The *Sox8* gene and associated *Sox8OT* ncRNA share a dynamically modified chromatin domain. The genome browser view shows the bidirectional organization of the *Sox8* gene (blue) and the *Sox8OT* ncRNA (red). A shared promoter region (shaded box) exhibits dynamic chromatin remodeling in embryonic stem cells (**A**; ES), embryonic fibroblasts (**B**; MEF) and neural progenitors (**C**; NP) [61]. The combination of active H3K4me3 (green histogram) and repressive H3K27me3 (red histogram) modified chromatin, observed in ES and MEF cells, is termed a bivalent domain and is indicative of a gene in a 'poised' state for activation. This is reflected in the low prevalence of H3K36me3 modified chromatin (blue histogram) that is normally associated with RNA Polymerase elongation. In NPs the bivalent domain has been resolved to an active H3K4me3 domain and the presence of H3K36me3 modified domains implies that both the *Sox8* and *Sox8OT* transcripts are concordantly upregulated. Tpm; tags per million.
