# Supplementary material for: Long noncoding RNAs in neuronal-glial fate specification and oligodendrocyte lineage maturation
Source: BMC Neurosci. 2010 Feb 5;11:14. doi: 10.1186/1471-2202-11-14 (PMC2829031; doi:10.1186/1471-2202-11-14)
Supplement: Additional file 15 — The HDAC inhibitor TSA prevents the acquisition of secondary morphological features of differentiating PMOs with concurrent alteration in the expression profiles of ncRNAs under instructive conditions (CNTF treatment) and stochastic (CNTF naïve) for OL lineage maturation. (A-D) Immunofluorescence micrographs demonstrating the profiles of OL lineage species in the absence (A, B) or presence (C, D) of TSA at T1 (24 h, A, C) and T2 (48 h, B, D) in the presence of CNTF with concurrent PDGF-AA factor withdrawal. (E, F) The effects of TSA application on comparative expression profiles of ncRNAs as assessed at T1 (E) and at T2 (F) in relation to the timing of TSA exposure in the experimental conditions. (G-K) Immunofluorescence micrographs demonstrating the profiles of OL lineage species in the absence (G, H) or presence (J, K) of TSA at T1 (24 h, G, J) and T2 (48 h, H, K) in the presence PDGF-AA factor withdrawal only. (L, M) The effects of TSA application on comparative expression profiles of ncRNAs assessed at T1 (L) and at T2 (M) in relation to the timing of TSA exposure in the experimental conditions. [file 1471-2202-11-14-S15.PDF]

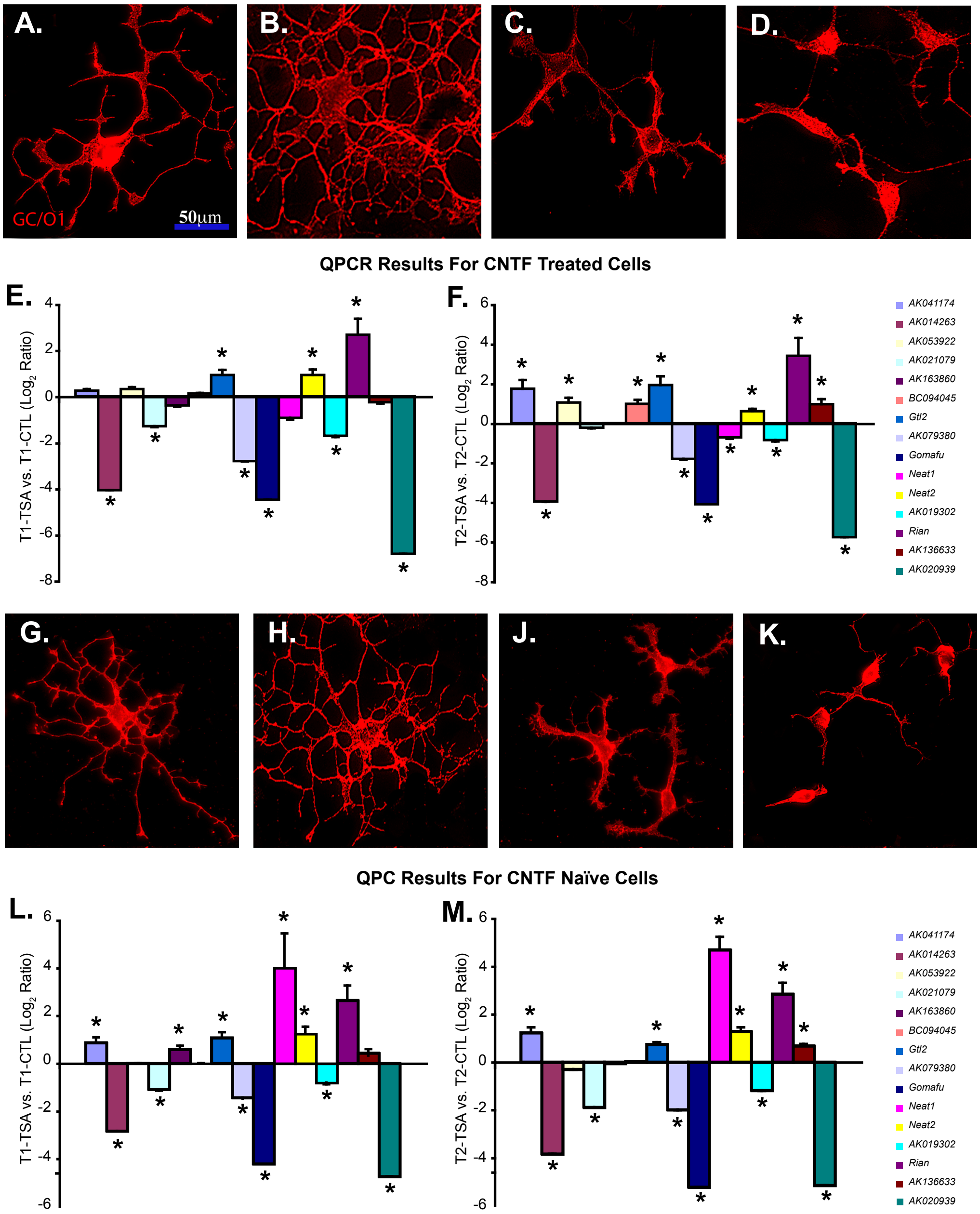

Additional File 15. The HDAC inhibitor TSA prevents the acquisition of secondary morphological features of differentiating PMOs with concurrent alteration in the expression profiles of ncRNAs under instructive conditions (CNTF treatment) and stochastic (CNTF naïve) for OL lineage maturation. (A-D) Immunofluoresence micrographs demonstrating the profiles of OL lineage species in the absence **(A, B)** or presence **(C, D)** of TSA at T1 (24 h., **A, C**) and T2 (48 h., **B, D**) in the presence of CNTF with concurrent PDGF-AA factor withdrawal. **(E, F)** The effects of TSA application on comparative expression profiles of ncRNAs as assessed at T1 **(E)** and at T2 **(F)** in relation to the timing of TSA exposure in the experimental conditions. **(G-K)** Immunofluoresence micrographs demonstrating the profiles of OL lineage species in the absence **(G, H)** or presence **(J, K)** of TSA at T1 (24 h., **G, J**) and T2 (48 h., **H, K**) in the presence PDGF-AA factor withdrawal only. **(L, M)** The effects of TSA application on comparative expression profiles of ncRNAs as assessed at T1 **(L)** and at T2 **(M)** in relation to the timing of TSA exposure in the experimental conditions.
